# (Epi)genetic variation in ageing of metabolic fitness

**DOI:** 10.1186/2049-3258-73-S1-P38

**Published:** 2015-09-17

**Authors:** Maarten Caspers, Ruben Charlier, Sara Knaeps, Evelien Mertens, Diether Lambrechts, Johan Lefevre, Katrien De Bock, Martine Thomis

**Affiliations:** 1 KU Leuven, Leuven, Belgium; 2 Vrije Universiteit Brussel, Brussels, Belgium

## 

Ageing-related diseases are an important cause of mortality. Although the susceptibility to these diseases is partially genetically determined, a person's physical activity level can influence the development of harmful alterations in the body through epigenetic pathways. Therefore, it is important to investigate both genetic and epigenetic aspects of ageing-related diseases. In this study, the focus lies on variables related to metabolic fitness: obesity, blood pressure, plasma glucose, serum triglycerides and high-density lipoproteins.

In the genetic part, a Genetic Predisposition Score (GPS) is calculated for metabolic fitness. This GPS is based on 28 selected single nucleotide polymorphisms (SNPs) and 54 expression quantitative trait loci (eQTLs) related to the metabolic fitness variables. In this context, DNA from blood samples of 842 adults of all age categories, measured within the framework of the Flemish Policy Research Centre Sport, was genotyped using the Illumina GoldenGate Genotyping Assay. The aim of the GPS is to determine the role of sequence variation in the interindividual variation in ageing regarding the metabolic fitness variables. Also the physical activity level of the subjects, assessed using the Flemish Physical Activity Computerized Questionnaire (FPACQ), will be included as covariate.

In the epigenetic part, about 70 men (born in 1954-1957), whose DNA is also genotyped, will be included in a longitudinal DNA methylation study. To determine the plasticity of DNA methylation over a 10-year period (2002-2012), the Illumina Infinium HumanMethylation450 BeadChip Assay will be used. The hypothesis regarding a first exploration is that less physically active people are subjected to more overall DNA demethylation during ageing, resulting in a stronger deterioration of metabolic fitness.

By combining information about sequence variation, DNA methylation plasticity and physical (in)activity, new insights can be acquired about the influence of both genetic factors and an (in)active lifestyle on metabolic fitness.

